# Factors associated with high-risk low-level viremia leading to virologic failure: 16-year retrospective study of a Chinese antiretroviral therapy cohort

**DOI:** 10.1186/s12879-020-4837-y

**Published:** 2020-02-17

**Authors:** Tong Zhang, Haibo Ding, Minghui An, Xiaonan Wang, Wen Tian, Bin Zhao, Xiaoxu Han

**Affiliations:** 1 NHC Key Laboratory of AIDS Immunology (China Medical University), Department of Laboratory Medicine, The First Affiliated Hospital of China Medical University, No 155, Nanjing North Street, Heping District, Shenyang, 110001 Liaoning Province China; 2 National Clinical Research Center for Laboratory Medicine, The First Affiliated Hospital of China Medical University, Shenyang, 110001 China; 3 Key Laboratory of AIDS Immunology of Liaoning Province, The First Affiliated Hospital of China Medical University, Shenyang, 110001 China; 4 Key Laboratory of AIDS Immunology, Chinese Academy of Medical Sciences, Shenyang, 110001 China; 5 Collaborative Innovation Center for Diagnosis and Treatment of Infectious Diseases, 79 Qingchun Street, Hangzhou, 310003 China

**Keywords:** HIV-1, Viral load assay, Low-level viremia, Virologic failure, Long-term antiretroviral therapy, First-line regimen, Lower-income countries

## Abstract

**Background:**

Low level viremia (LLV) often occurs during antiretroviral therapy (ART) against HIV-1. However, whether LLV increases the risk of virologic failure (VF) is controversial because of the non-uniform definitions of LLV and VF.

**Methods:**

A long-term first line regimen ART cohort from 2002 to 2018 from Shenyang, northeast China, was retrospectively studied. All participants were followed up every 3 to 6 months to evaluate the treatment effect. The high-risk LLV subgroups leading to VF (with strict standards) were explored with Cox proportional hazards model and linear mixed-effect model. The association factors of high-risk LLV were further explored using multivariate logistic regression analyses.

**Results:**

A total of 2155 HIV-1 infected participants were included; of these, 38.7% showed LLV. Both high level LLV (HLLV) and any other level LLV coupled with high level blip (HLB) showed higher risk of VF (hazards ratios, HR_HLLV_ = 5.93, and HR_HLB_ = 2.84, *p* <  0.05 respectively). Moreover, HR increased with prolonged duration of LLV. Independent factors associated with high-risk LLV included the zenith baseline viral load (VL) above 6 log copies/ml (aOR = 3.49, *p* = 0.002), nadir baseline CD4 + T cell counts below 200 cells/mm^3^ (aOR = 1.78, *p* = 0.011), Manchu (aOR = 2.03, *p* = 0.003), ART over 60 months (aOR = 1.81, *p* = 0.004), AZT + 3TC + NVP (aOR = 2.26, *p* <  0.001) or DDI-based regimen (aOR = 9.96, *p* = 0.002), and subtype B′ infection (aOR = 8.22, *p* = 0.001).

**Conclusions:**

In case of VF with strict standards, high-risk LLV leading to VF includes VL above 400 copies/ml, occurring at least once. Serious laboratory indicators or advanced stage of infection, long term ART and subtype B′ infection might also predict the occurrence of high-risk LLV.

## Background

Human immunodeficiency virus type 1 (HIV-1) rapidly replicates mainly in CD4 + T cells and is released into the blood. Antiretroviral therapy (ART) can limit virus replication to less than 50 copies/ml and effectively prolong the life of HIV-1 infected patients [1]. However, in approximately 10–30% of the patients, depending on the definition, standard ART cannot control viral replication completely [2, 3]. The plasma of such patients shows low level of viral replication, which is designated as low level viremia (LLV) [4]. Among these cases, the occasionally detected viremia is designated as viral blip (Blip) [5], whereas the continuously detected viremia is designated as persistent low level viremia (pLLV) [6]. The standards of LLV vary greatly with different guidelines. In resource-limited countries or regions [2, 7, 8], LLV is defined as a viral load (VL) between 50 and 1000 copies/ml, according to the World Health Organization (WHO) guidelines [9]. In United States and some other resource-rich countries [10–12], LLV is defined as a VL between 50 and 200 copies/ml according to the Department of Health and Human Services (DHHS) guidelines (USA, 2016) [13]. In Europe and most resource-rich settings [14, 15], LLV is defined as a VL between 20 and 50 copies/ml, according to the European acquired immune deficiency syndrome (AIDS) Clinical Society (EACS) guidelines [16]. Although LLV frequently occurs during treatment, none of the guidelines suggest changing the treatment regimen or intensifying treatment in case of LLV.

The impact of LLV on clinical prognosis is still controversial. An earlier national observational cohort study from USA found that an LLV above 400 copies/ml could not predict AIDS or death [17]. However, multiple recent cohort studies worldwide demonstrated that the level of LLV is a determinant of prognosis; high level of LLV (> 200 copies/ml) occurring only once might increase the risk of virologic failure (VF) [7, 14, 18]. Some studies from developed countries found that persistent LLV or frequently occurring LLV of 50–200 copies/ml also increased the risk of VF [6, 19, 20]. However, a recent multicenter study from South Africa found that LLV below 200 copies/ml, whether continuous or not, contributed to increased VF risk [2]. Moreover, several recent studies found that even very low levels of pLLV between 20 and 50 copies/ml could predict an increased risk of viral failure [14, 21]. Most of the above studies varied in their standards of LLV or VF definitions, and in some studies, the definition of VF was not stringent. For example, a recent multicenter cohort study reported that approximately 20% VF patients, according to the standard of WHO guideline, could spontaneously control viral load to below 1000 copies/ml during the follow up time without any intervention; however, these cases did not strictly belong to the VF category [2]. Moreover, no study has evaluated the impact of LLV on the risk of VF considering both level and duration of LLV.

Although most studies suggest that LLV contributes to VF, the potential risk factors of LLV have not been sufficiently investigated. Several HIV cohort studies from developed countries where subtype B was predominant found that the high zenith baseline VL and low nadir baseline CD4 + T cell counts were associated with the risk of LLV [3, 22–25]. However, the evidence from non-subtype B infected cases and resource-limited regions are still lacking.

The aims of the current study, which used clinical data from a long-term first line regimen ART cohort in China, were to determine the characteristics of high-risk LLV associated with VF, and to elucidate the factors contributing to this kind of LLV, by considering both the level and the duration of LLV. The results of this study might provide new evidence for the occurrence and contribution of risk factors of LLV, and thereby help to optimize ART treatment in resource-limited setting.

## Methods

### Study population

This retrospective study was based on an open HIV-1 infection treatment cohort in a big comprehensive hospital in Shenyang, northeastern China between 2002 and 2018. All subjects received first-line ART regimen according to Chinese guidelines for diagnosis and treatment of HIV/AIDS [26]. This included two nucleoside reverse transcriptase inhibitors (NRTIs) and a non- nucleoside reverse transcriptase inhibitor (NNRTI) that are widely used globally [27–31]. All participants were followed up every 3 to 6 months for evaluation of VL, CD4 + T cell counts, and other routine clinical parameters. The socio-demographic information included gender, age at HIV diagnosis (< 50 years), marital status, education, ethnicity, steady income, and transmission route. The clinical data, including duration on ART (months), ART regimen, and laboratory test results including VL, CD4 + T cell counts, and HIV genotype, were collected from clinical follow-up case records.

The inclusion criteria for this study were as follows: (1) participants received first-line ART regimen in China (2 NRTIs and 1 NNRTI); (2) VL had been controlled to less than 50 copies/ml after 6 months treatment; (3) participants were followed-up for at least 12 months. Patients were excluded from this study if their VLs were incomplete. This study was approved by the ethics committee of the First Affiliated Hospital of China Medical University. In addition, informed consents were obtained from all participants.

### Definitions of LLV and VF

In this study, we used the definition of LLV based on the WHO guideline [9]; LLV was defined as viremia between 50 and 1000 copies/ml during ART, and was classified into three groups: high level LLV of 400–1000 copies/ml (HLLV), medium level LLV of 200–400 copies/ml (MLLV), and low level LLV of 50–200 copies/ml (LLLV). Any level LLV coupled with a high level blip of above 1000 copies/ml was defined as high level blip (HLB). The definition of VF was modified on the basis of the WHO standard to two or more consecutive VL of above 1000 copies/ml and viral replication that could not be controlled spontaneously without changing therapeutic regimens.

### Statistical analyses

The CD4 lymphocyte count and the results of viral load were included as quantitative variables. The K-S normal test method was used to evaluate the normality of variables. The comparisons were made between LLV categories by log-rank tests. Chi-squared test was used for comparisons among other variables. The incidence of VF was calculated by the total number of VF occurrence/total follow-up time * 100% [32, 33]. The impacts of LLV on VF were evaluated by the Cox proportional hazards model and the linear mixed-effect model (shown with hazard ratio (HR) and estimate with their 95%CI), in which VL were analyzed as time-varying covariates [7]. The characteristics of high risk LLV that increased the risk of subsequent VF were determined. Multiple factors, consisting of socio-demographic information, transmission route, clinical data, and laboratory test results, were used to identify potential factors of high-risk LLV by multivariate logistic regression, with correction for sex and age [34]. Survival analysis was performed to calculate the association between all LLV categories and VF. Kaplan-Meier curves were created for the entire study population according to the level and duration of LLV. In creating the survival curves, time to event (i.e. VF) was estimated from the date of ART initiation to the date of VF; in absence of VF, we used the date of last clinic visit as the follow-up time. All statistical analyses were conducted using Statistical Package for the Social Sciences software (SPSS 22.0) and a *p*-value of less than 0.05 was considered statistically significant.

## Results

### Characteristics of participants in this study

**Fig. 1:**
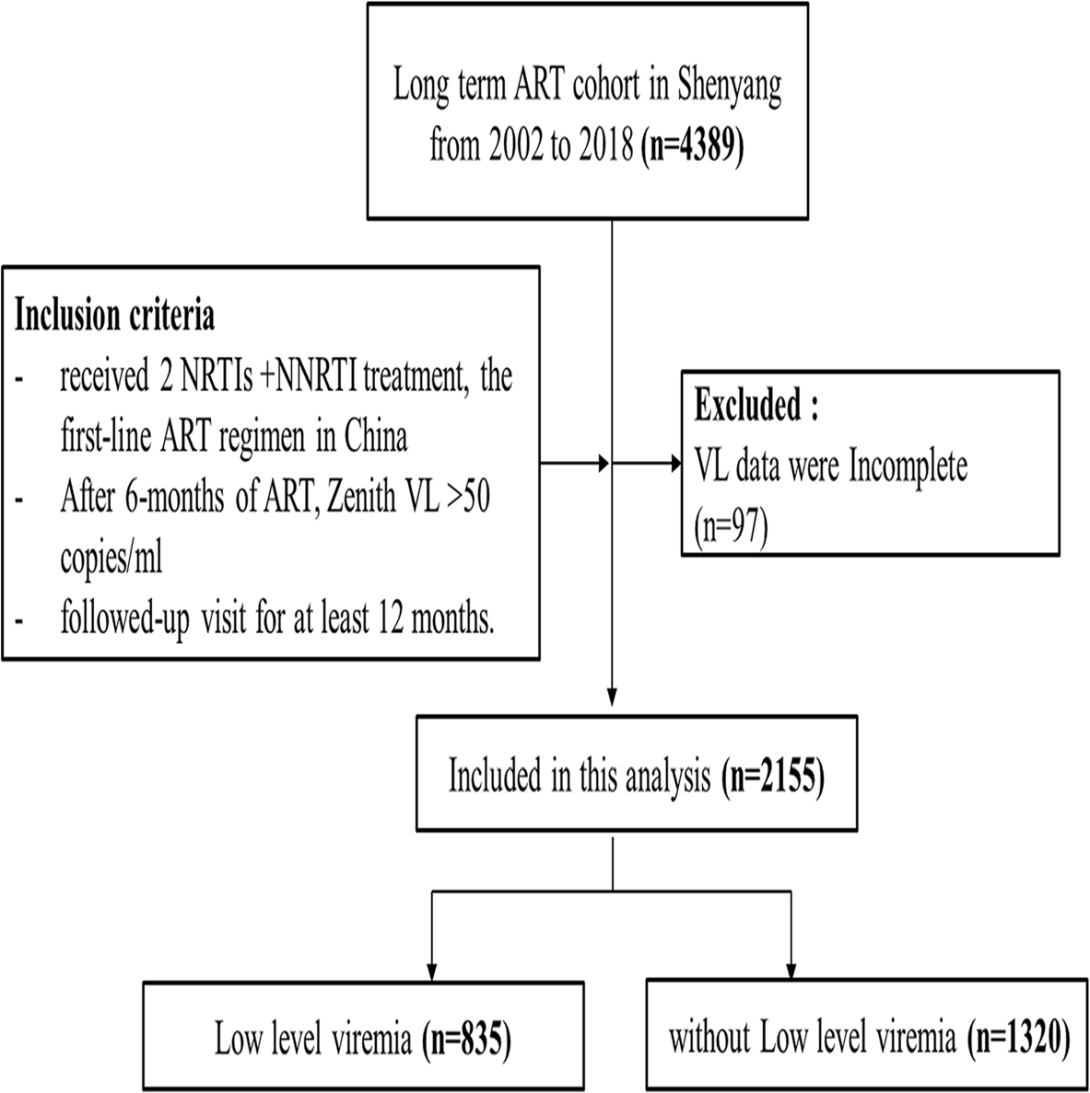
Flowchart showing selection of the participants

In total, 2155 participants were selected from a long-term, first-line ART regimen, local Chinese cohort (Fig. 1). The patients in this study were followed up to 8348 person-years in total, and with the median follow up time of 3.4 years (IQR: 2.3–5.0). The majority of the participants were male (92.9%, 2001/2155), with a median age at HIV diagnosis of 37 years old (IQR: 31–49). They mainly comprised of the Han nationality (85.4%). Further, 79.2% (1706/2155) of the participants were infected with HIV-1 through homosexual transmission, 65.1% (1403/2155) were infected with CRF01_AE HIV-1, and 68.5% (1477/2155) of participants used TDF + 3TC + EFV as the ART regimen. Most participants (50.9%, 1096/2155) showed the zenith baseline VL of 4–5 (log copies/ml) and 37.4% (805/2155) of participants displayed the nadir baseline CD4 + T cell counts of 200–350 cells/mm^3^ (Additional file 1: Table S1).

In this study, 38.7% (835/2155) of participants experienced at least one episode of LLV. In terms of the LLV level, participants belonging to LLLV, MLLV, HLLV, and HBL groups accounted for 52.1% (435/835), 18.0% (150/835), 14.3% (119/835), and 15.7% (131/835), respectively. In terms of the duration of LLV, participants with Blip, LLV lasting for 3–6 months, 6–12 months, and above 12 months accounted for 72.1% (602/835), 11.4% (95/835), 11.4% (95/835), and 5.1% (43/835), respectively (Additional file 1: Table S1).

### High risk LLV contributes to VF

**Fig. 2:**
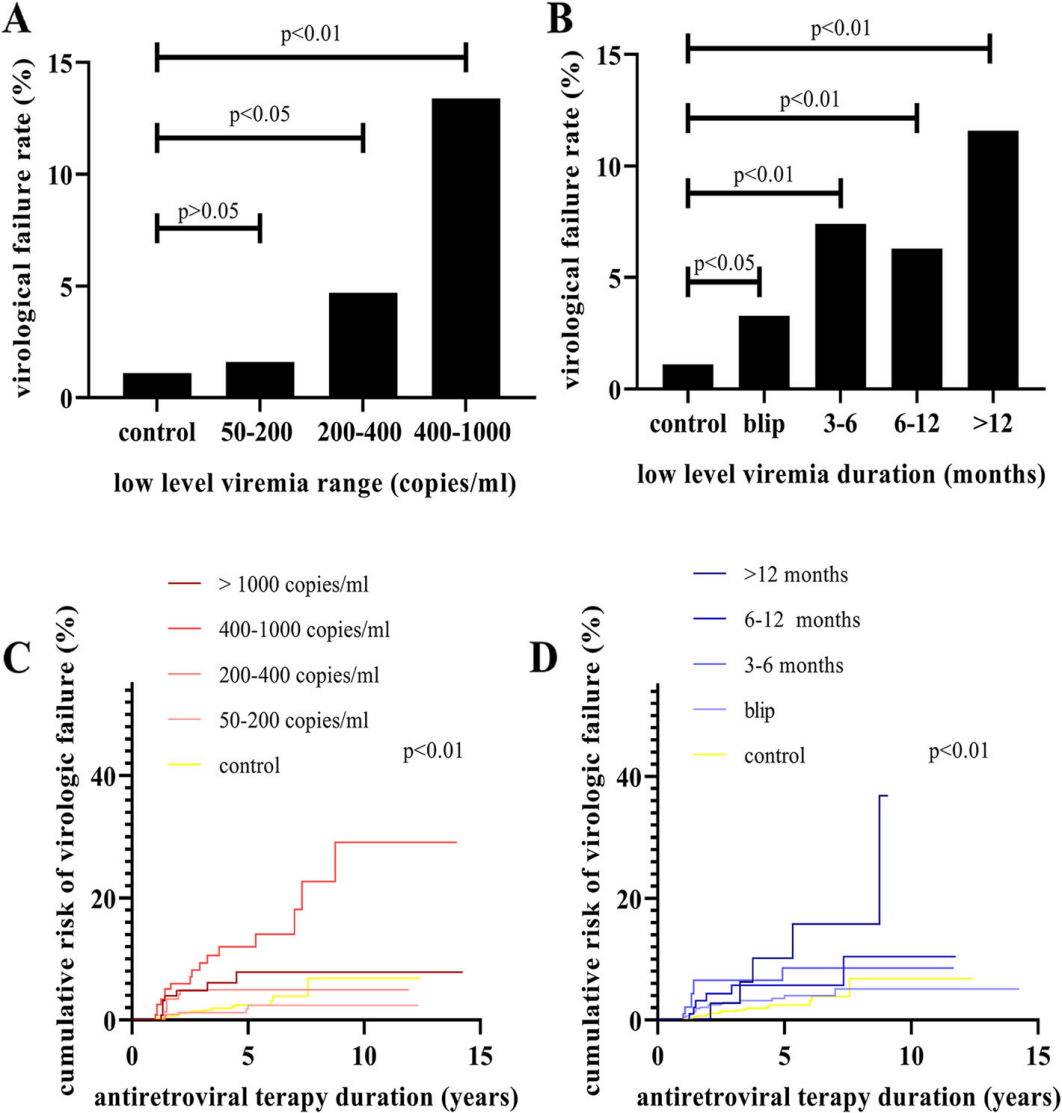
Incidence of virologic failure according to the level and the duration of low level viremia (LLV). Incidence of virologic failure according to the (**a**) level of LLV, and (**b**) duration of LLV. Kaplan-Meier estimate for virologic failure according to the (**c**) level of LLV, and (**d**) duration of LLV

The VF rate increased along with increasing level of LLV (50–200 copies/ml: 1.6%, 200–400 copies/ml: 4.7%, 400–1000 copies/ml: 13.4%, Fig. 2a) and the duration of viremia (Blip: 3.3%, 3–6 months: 7.4%, 6–12 months: 6.3%, > 12 months: 11.6%, Fig. 2b). On the basis of the estimated Kaplan–Meier curves, there were significant differences in the risk of VF (*p* <  0.01) between LLV level and duration categories. Compared with the control group (incidence rate (IR): 0.52/100 person years), those with > 400 copies/ml (IR: 2.97/100 person years, *p* <  0.01) and LLV duration of > 12 months (IR: 3.68/100 person years, *p* <  0.01) had the least favorable Kaplan–Meier curves and higher risk of VF (Fig. 2c, d). The Cox proportional hazards model showed that only HLLV (HR: 5.93, 95%CI: 3.13–11.23, *p* <  0.01) and HBL (HR: 2.84, 95%CI: 1.27–6.34, *p* = 0.011) were associated with significantly increased risk of subsequent VF compared with the control group (all VL below 50 copies/ml).

Based on LLV duration analyses, we found that the risk of VF increased in the HLLV group for any duration; moreover, the HRs increased with prolonged duration (Blip: HR = 4.41, 95%CI: 1.96–9.92, *p* < 0.01; 3–6 months: HR = 6.59, 95%CI: 1.94–22.34, *p* < 0.01; 6–12 months: HR = 5.19, 95%CI: 1.22–22.18, *p* < 0.05; and >  12 months: HR = 9.63, 95%CI: 2.82–32.95, *p* < 0.01). In addition, any level LLV (> 3 months) coupled with HBL also showed increased risk of VF (3–6 months: HR = 6.20, 95%CI: 1.46–26.27, *p* < 0.05; 6–12 months: HR = 9.74, 95%CI: 2.82–33.66, *p* < 0.01; > 12 months: HR = 7.52, 95%CI: 1.01–55.84, *p* < 0.05). In contrast, no significant increase of VF was observed among MLLV and LLLV groups, even in those that lasted for > 12 months (200–400 copies/ml: HR = 3.43, 95%CI: 0.46–25.54, *p* > 0.05; 50–200 copies/ml: HR = 1.02, 95%CI: 1.01–1.03, *p* > 0.05).

In this study, VL was determined at 3–6 months interval and acted as a time-varying covariate. We further used the linear mixed-effect model to validate the contribution of LLV on VF [35, 36]. Our results supported the contribution of HLLV and HLB to VF, but the MLLV group showed weak association with increased risk of VF (estimated 0.03, 95%CI: 0.00–0.06, *p* = 0.045). LLLV showed no increase of VF in both models (Table 1).

**Table 1:** Association between LLV characteristics and virologic failure in the linear mixed-effect model and the Cox proportional hazards model (*N* = 2155)

LLV group^a^	Case Number	Cox proportional hazards model		Linear mixed-effect model	
		HR (95%CI)	*p*-value	Estimate (95%CI)	*p*-value
< 50 (copies/ml)	1320	1(reference)		0(reference)	
50–200 (copies/ml)	435	0.72(0.31–1.68)	0.453	0.00(− 0.02–0.02)	0.819
blip^b^	318	0.68(0.26–1.80)	0.438	0.00(− 0.02–0.01)	0.765
3–6 (months)^c^	47	1.58(0.37–6.80)	0.541	0.02(−0.01–0.06)	0.213
6–12 (months)^d^	53	1.02(1.01–1.03)	0.975	−0.02(− 0.05–0.02)	0.325
> 12 (months)^e^	17	1.02(1.01–1.03)	0.986	−0.02(− 0.08–0.04)	0.572
200–400 (copies/ml)	150	1.90(0.81–4.43)	0.139	**0.03(0.00–0.06)**	**0.045**
blip^b^	108	1.91(0.72–5.06)	0.196	0.03(0.00–0.06)	0.051
3–6 (months)^c^	14	1.02(1.01–1.03)	0.979	−0.02(−0.10–0.06)	0.637
6–12 (months)^d^	17	2.12(0.28–15.99)	0.468	0.04(−0.03–0.11)	0.246
> 12 (months)^e^	11	3.43(0.46–25.54)	0.229	0.07(−0.01–0.16)	0.094
**400–1000 (copies/ml)**	119	**5.93(3.13–11.23)**	**0.000**	**0.12(0.09–0.15)**	**0.000**
**blip**^**b**^	78	**4.41(1.96–9.92)**	**0.000**	**0.08(0.05–0.12)**	**0.000**
**3–6 (months)**^**c**^	18	**6.59(1.94–22.34)**	**0.002**	**0.15(0.07–0.22)**	**0.000**
**6–12 (months)**^**d**^	14	**5.19(1.22–22.18)**	**0.026**	**0.12(0.04–0.21)**	**0.004**
**> 12 (months)**^**e**^	9	**9.63(2.82–32.95)**	**0.000**	**0.32(0.21–0.42)**	**0.000**
**HLB > 1000 (copies/ml)**	131	**2.84(1.27–6.34)**	**0.011**	**0.04(0.01–0.07)**	**0.005**
blip^b^	98	0.97(0.23–4.09)	0.961	0.00(−0.03–0.03)	0.883
**3–6 (months)**^**c**^	16	**6.20(1.46–26.27)**	**0.013**	**0.11(0.04–0.19)**	**0.003**
**6–12 (months)**^**d**^	11	**9.74(2.82–33.66)**	**0.000**	**0.25(0.17–0.34)**	**0.000**
**> 12 (months)**^**e**^	6	**7.52(1.01–55.84)**	**0.049**	**0.15(0.03–0.27)**	**0.012**

a: LLV group is defined according to the Zenith VL during ART, subgroup is further divided according to the duration of LLV at any level between 50 and 1000 copies/ml. b: single viral blip, c: pLLV lasts 3 months, d: pLLV lasts 6 months, e: pLLV lasts more than 12 months. *VL* viral load, *HR* hazard ratio, *CI* confidence interval, *HLB* high level blip. The bold data in the table shows the subgroup with increased risk of the subsequent VF, which we defined as high-risk LLV

### Factors associated with high risk LLV

We further assessed the socio-demographic information, transmission route, clinical data, and laboratory test results to explore the factors associated with high risk LLV. Using chi-square tests, we found that the different groups of zenith baseline VL, nadir CD4 + T cell counts, and other demographic information, clinical data, and laboratory tests had varied frequencies of high risk LLV (Table 2). Multiple logistic regression analysis further supported that participants with higher zenith baseline VL (> 6 log copies/ml vs. < 4 log copies/ml: aOR = 3.49, 95%CI: 1.55–7.83, *p* < 0.01) and lower nadir baseline CD4 + T cell counts (< 200 cells/mm^3^ vs. > 350 cells/mm^3^: aOR = 1.78, 95%CI: 1.14–2.78, *p* < 0.05) showed increased risk of high-risk LLV. Other associated factors included Manchu (aOR: 2.03, 95%CI: 1.27–3.25, *p* < 0.01), ART for over 60 months (aOR: 1.81, 95%CI: 1.21–2.69, *p* < 0.01), receiving AZT + 3TC + NVP regimen (aOR: 2.26, 95%CI: 1.48–3.45, *p* < 0.01) or DDI + NRTI+NNRTI regimen (aOR: 9.96, 95%CI: 2.33–42.49, *p* < 0.01) and subtype B′ infection (aOR: 8.22, 95%CI: 2.34–28.92, *p* < 0.01).

**Table 2:** Factors associated with high risk LLV

Characteristics	Case Number	High risk LLV case number (%)	*p*-value	Unadjusted analysis		Adjusted analysis	
				OR(95%CI)	*p*-value	aOR(95%CI)	*p*-Value
Gender							
Male	2001	142(7.10)	0.957	1.00(reference)			
Female	125	8(6.40)		1.03(0.24–4.38)	0.967		
NA	29	2(6.90)		0.92(0.19–4.60)	0.922		
Age at HIV diagnosis							
≤ 50 (years)	1618	110(6.80)	0.607	1.00(reference)			
> 50 (years)	500	40(8.00)		0.78(0.19–3.30)	0.739		
NA	37	2(5.41)		1.19(0.82–1.74)	0.361		
Nation							
Han	1840	123(6.68)	0.017	1.00(reference)		1.00(reference)	
Manchu	195	24(12.31)		1.96(1.23–3.12)	0.005	2.03(1.27–3.25)	0.003
Others	82	3(3.66)		0.53(0.17–1.70)	0.287	0.55(0.17–1.76)	0.310
NA	38	2(5.26)		0.78(0.19–3.26)	0.729	0.60(0.10–3.64)	0.579
Duration on ART							
12–36(months)	954	57(5.97)	0.004	1.00(reference)		1.00(reference)	
36–60(months)	674	41(6.08)		1.02(0.67–1.54)	0.928	1.02(0.67–1.54)	0.925
> 60(months)	527	54(10.25)		1.80(1.22–2.65)	0.003	1.81(1.21–2.69)	0.004
ART regimen							
TDF + 3TC + EFV	1477	84(5.69)	< 0.001	1.00(reference)		1.00(reference)	
AZT + 3TC + EFV	276	20(7.25)		1.30(0.78–2.15)	0.315	1.31(0.79–2.18)	0.301
AZT + 3TC + NVP	294	35(11.90)		2.24(1.48–3.40)	< 0.001	2.26(1.48–3.45)	< 0.001
D4T + 3TC + EFV	41	5(12.20)		2.30(0.88–6.02)	0.089	2.38(0.90–6.33)	0.082
D4T + 3TC + NVP	59	5(8.47)		1.54(0.60–3.94)	0.372	1.56(0.61–4.04)	0.355
DDI + a NRTI +a NNRTI	8	3(37.50)		9.95(2.34–42.34)	0.002	9.96(2.33–42.49)	0.002
Transmission route							
Sexual	1965	132(6.72)	0.133	1.00(reference)		1.00(reference)	
Others	24	2(8.33)		1.26(0.29–5.43)	0.754	1.31(0.30–5.67)	0.715
NA	166	18(10.84)		1.69(1.00–2.84)	0.048	1.84(1.05–3.23)	0.034
HIV-1 subtype							
B	130	7(5.38)	0.001	1.00(reference)		1.00(reference)	
B′	20	6(30.00)		7.53(2.22–25.57)	0.001	8.22(2.34–28.92)	0.001
CRF01_AE	1403	99(7.06)		1.33(0.61–2.94)	0.474	1.32(0.60–2.90)	0.495
CRF07_BC	213	19(8.92)		1.72(0.70–4.21)	0.235	1.73(0.71–4.23)	0.232
Others	95	4(4.21)		0.77(0.22–2.72)	0.687	0.77(0.22–2.73)	0.690
Na	294	17(5.78)		1.08(0.44–2.67)	0.870	1.09(0.44–2.71)	0.849
Zenith baseline VL							
< 4 (log_10_ copies/ml)	535	30(5.61)	0.006	1.00(reference)		1.00(reference)	
4–5 (log_10_ copies/ml)	1096	72(6.57)		1.18(0.76–1.84)	0.452	1.18(0.76–1.83)	0.469
5–6 (log_10_ copies/ml)	472	41(8.69)		1.60(0.98–2.61)	0.059	1.58(0.96–2.58)	0.070
> 6 (log_10_ copies/ml)	52	9(17.31)		3.52(1.57–7.90)	0.002	3.49(1.55–7.83)	0.002
Nadir baseline CD4+ cell counts							
> 350 (cells/ml)	665	34(5.11)	0.058	1.00(reference)		1.00(reference)	
200–350 (cells/mm^3^)	805	57(7.08)		1.41(0.91–2.19)	0.121	1.41(0.91–2.19)	0.123
< 200 (cells/mm^3^)	634	56(8.83)		1.80(1.16–2.79)	0.009	1.78(1.14–2.78)	0.011
NA	51	5(9.80)		2.02(0.75–5.40)	0.163	2.03(0.76–5.43)	0.160

Adjusted for gender and age

*LLV* low level viremia, participants with VL results range of > 50 copies per ml but not defined as virologic failure, *OR* odds ratio, *aOR* adjusted odds ratio, *CI* confidence interval, *NA* Not available, *ART* antiretroviral therapy, *HIV* human immunodeficiency virus, *TDF* Tenofovir, *3TC* Lamivudine, *EFV* Efavirenz, *AZT* Zidovudine, *NVP* Nevirapine, *D4T* Stavudine, *DDI* didanosine, *NRTIs* nucleotide analogue reverse transcriptase inhibitors, *NNRTIs* nonnucleotide Analogue Reverse Transcriptase Inhibitors

## Discussion

In this study, we observed LLV from 38.7% of participants in a local long-term ART cohort from northeastern China. With a strict definition of VF, we found that the subgroup of high level LLV of above 400 copies/ml and any level LLV coupled with blip of above 1000 copies/ml were high-risk LLV associated with VF. Moreover, we found that the duration of LLV also played an important role in contributing to the risk of VF. The association factors included high zenith VL, low nadir CD4 + T cell counts, long term ART, Manchu, and subtype B′ infection.

The occurrence of LLV has not been reported in China, where uniform ART is provided freely; however, there is a lack of individual monitoring and treatment. In this study, we observed LLV from 38.7% of participants, and this was much higher than the LLV occurrence (15–20%) in resource-rich settings with similar LLV definition criteria (50–1000 copies/ml) [18, 37]. Multiple potential factors could explain these differences, including different ART regimens and frequency of testing [6]. However, because of the strict definition of VF used in this study, the occurrence of VF was substantially lower than previous reported data on both resource-rich regions [38, 39] and resource-limited regions [2, 7]. In a recent study from South Africa, 26% VF according to standard of WHO (VL > 1000 copies/ml once) showed spontaneous control of viral replication without changing treatment regimen, and therefore should not be considered as true VF [2]. In this study, we adjusted the standard of VF to two or more consecutive VL of above 1000 copies/ml with more than a 3-month interval. This new definition can avoid unnecessary ART regimen switches [2, 4], which is very important for resource-limited regions.

Using this new VF definition, we revealed that both level and duration of LLV were factors associated with VF. In this study, only LLV above 400 copies/ml could increase the risk of VF, which differed from several recent studies that implied a load of as low as 20 copies/ml might lead to VF [15, 21]. Such findings are usually obtained from source-rich regions, where abundant drugs are available and a completely different standard of VF (50 copies/ml) is used; therefore, this is not necessarily applicable in most resource-limited regions. Moreover, most previous studies have only evaluated the impact of different level LLV on VF, without considering the duration of LLV. A few studies found no significant association of low or medium levels LLV with varied duration [5, 6, 38]. However, the definition of VF in these studies was two consecutive viral loads of at least 500 copies/ml, which is rarely used in practice and differed from that used in this study.

Moreover, we also found that any level of LLV coupled with HLB increased the VF risk. In previous studies, HLB (> 1000 copies/ml) was usually defined as VF. However, in our study, 6.1% (131/2155) participants (with average VL of 12,741 copies/ml) showed spontaneous control of viral replication in the follow up time. This phenomenon is more common in the cohort of South Africa (26%), which we speculate is caused by irregular compliance or accident [2]. Previous reports indicated that MLLV is associated with increased occurrence of VF [19, 20]; however, in the current study, we observed only an increasing trend of VF if lasting for 12 months. Thus, this potential association needs further validation.

Our current study implied that MLLV or LLLV were not associated with higher risk of VF, and therefore such cases should be only kept under observation without changing treatment. In contrast, a previous study suggested that patients with such level of LLV should be switched to other regimes or given stronger treatments immediately.

Some previous studies have considered both level and duration of LLV, which introduced a new parameter of viremia copy-years (VCY), combining the level and duration of virus replication, defined as the area under a patient’s longitudinal VL curve. These studies found that the risk of VF rose with increasing VCY, which is consistent with the findings of the current study. However, no uniform threshold had been determined, because of the varied definitions of both LLV and VF [24, 25]. In contrast to previous studies, we focused only on the factors of high-risk LLV subgroup leading to VF, to identify conditions that need prompt treatment during ART. Although we used new definitions of LLV and VF, we found some similar risk factors as several resource-rich cohort studies, including the high zenith VL and low nadir CD4 + T cell counts [3, 22, 23]. In addition, we found that long-term ART, AZT + 3TC + NVP or DDI + NRTI+NNRTI regimens, Manchu and subtype B′ infection were all risk factors in this study. In the early stage of this cohort, DDI-based regimen was used as first-line regimens according to the guide at that time, although these regimens were stopped several years later. It have been reported that DDI-based regimen-treated patients showed a higher cumulative risk of drug resistance [40] and the AZT + 3TC + NVP regimen had long-term sequelae and toxic and side effects [41, 42]; these findings offered a rational explanation for the increased frequency of high-risk LLV in this study.

According to this study, for patients with the baseline characters, such as high zenith VL, low nadir CD4 + T cell counts, Manchu, and subtype B′, who were more likely to develop high-risk LLV, a more frequent follow-up should be recommended. The patients with LLV levels above 400 copies/ml, including both HLLV and HLB, and MLLV persisting longer than 12 months should be recommended to undergo drug resistance testing and blood drug concentration testing. On the other hand, for the patients with LLV levels below 200 copies/ml, follow-up can be continued.

Our study has some limitations. First, as a retrospective study, there may be some bias in participant selection. Second, 90% patients in this study self-reported good drug compliance, but the drug concentration in their plasma was not detected; thus, we were unable to assess the reliability of self-reported compliance. Third, about 5–10% of the social demographic and laboratory information were unavailable in this study, which might impact the results.

## Conclusion

In conclusion, we observed the common occurrence of LLV among a local long-term ART cohort in China, among which, some LLV demonstrated with higher risk of VF and should be treated immediately. Our study also identified prognostic factors that can help determine the potential occurrence of high risk LLV. The results of this study provide a basis for development of differential treatment of LLV at various levels.

## Supplementary information

**Additional file 1: Table S1.** Baseline characteristics of the included participants (*N* = 2155).

## Data Availability

The datasets used and/or analyzed during the current study are available from the corresponding author upon reasonable request.

## Abbreviations

3TC: Lamivudine
ART: Antiretroviral therapy
AZT: Zidovudine
Blip: Viral blip
CI: Confidence interval
D4T: Stavudine
DDI: Didanosine
DHHS: Department of Health and Human Services
EACS: European acquired immune deficiency syndrome Clinical Society
EFV: Efavirenz
HIV: Human immunodeficiency virus
HLB: High level blip
HLLV: High level LLV
HR: Hazard ratio
IQR: Interquartile range
IR: Incidence rate
LLV: Low level viremia
NA: Not available
NNRTI: Non-nucleoside reverse transcriptase inhibitor
NRTIs: Nucleoside reverse transcriptase inhibitors
NVP: Nevirapine
pLLV: Persistent low level viremia
TDF: Tenofovir
VCY: Viremia copy-years
VF: Virologic failure
VL: Viral load
WHO: World Health Organization
