# Supplementary material for: Factors associated with high-risk low-level viremia leading to virologic failure: 16-year retrospective study of a Chinese antiretroviral therapy cohort
Source: BMC Infect Dis. 2020 Feb 17;20:147. doi: 10.1186/s12879-020-4837-y (PMC7026956; doi:10.1186/s12879-020-4837-y)
Supplement: Supplementary file 1 — Additional file 1: Table S1. Baseline characteristics of the included participants (N = 2155). [file 12879_2020_4837_MOESM1_ESM.docx]

Table S1 Baseline characteristics of the included participants (N = 2155)

| Characteristics | Case number (%) |
| --- | --- |
| Socio-demographic information |  |
| Gender |  |
| Male | 2001(92.85) |
| Female | 125(5.80) |
| NA | 29(1.35) |
| Age at HIV diagnosis (<50 years) |  |
| < 50 | 1618(75.08) |
| ≥ 50 | 500(23.20) |
| NA | 37(1.72) |
| Marital status |  |
| married | 621(28.82) |
| single | 1225(56.84) |
| divorced | 252(11.69) |
| widow | 24(1.11) |
| NA | 33(1.53) |
| Education |  |
| < 7 years | 77(3.57) |
| 7–12 years | 912(42.32) |
| > 12 years | 1010(46.87) |
| NA | 156(7.24) |
| Ethnicity |  |
| Han | 1840(85.38) |
| Manchu | 195(9.05) |
| Others | 82(3.81) |
| NA | 38(1.76) |
| Steady income |  |
| Yes | 1476(68.49) |
| No | 566(26.26) |
| NA | 113(5.24) |
| Clinical data |  |
| Duration on ART (months) |  |
| 12–36 | 954(44.27) |
| 36–60 | 674(31.28) |
| > 60 | 527(24.45) |
| ART regimen |  |
| TDF+3TC+EFV | 1477(68.54) |
| AZT+3TC+EFV | 276(12.81) |
| AZT+3TC+NVP | 294(13.64) |
| D4T+3TC+EFV | 41(1.90) |
| D4T+3TC+NVP | 59(2.74) |
| DDI+1NRTI+1NNRTI | 8(0.37) |
| Transmission route |  |
| Heterosexual | 259(12.02) |
| Homosexual | 1706(79.16) |
| Others | 24(1.11) |
| NA | 166(7.70) |
| Laboratory test |  |
| HIV-1 subtype |  |
| B | 130(6.03) |
| B’ | 20(0.93) |
| CRF01_AE | 1403(65.10) |
| CRF07_BC | 213(9.88) |
| Other subtype | 95(4.41) |
| NA | 294(13.64) |
| Zenith baseline VL (log_10_ copies/ml) |  |
| < 4 | 535(24.83) |
| 4–5 | 1096(50.86) |
| 5–6 | 472(21.90) |
| > 6 | 52(2.41) |
| Nadir baseline CD4+ cell count (cells/mm^3^) |  |
| < 200 | 634(29.42) |
| 200–350 | 805(37.35) |
| > 350 | 665(30.86) |
| NA | 51(2.37) |
| LLV level (copies/ml)^a^ |  |
| 50-200 | 435(52.10) |
| 200-400 | 150(17.96) |
| 400-1000 | 119(14.25) |
| high level blip^b^: >1000 | 131(15.69) |
| LLV duration (months) |  |
| Blip | 602(72.10) |
| 3–6 | 95(11.38) |
| 6–12 | 95(11.38) |
| > 12 | 43(5.15) |

a, LLV level is defined according to the Zenith VL during ART.

b, participants occurred once or more intermittent viremia (blip) above 1000 copies/ml during ART.

NA, Not available; TDF, Tenofovir; 3TC, Lamivudine; EFV, Efavirenz; AZT, Zidovudine; NVP, Nevirapine; D4T, Stavudine; DDI, Didanosine; NRTIs, Nucleoside reverse transcriptase inhibitors; NNRTIs, non-nucleoside reverse transcriptase inhibitors; LLV, low level viremia.
